# SLEEP AND STRESS IN MIDDLE-AGED AND OLDER CAREGIVERS OF CHILDREN, PARENTS, AND SPOUSES: EXAMINING SEX DIFFERENCES

**DOI:** 10.1093/geroni/igae098.3153

**Published:** 2024-12-31

**Authors:** Melanie Stearns, Kevin McGovney, Ashley Curtis, Amy Costa, Mary Beth Miller, Uma Nair, Christina McCrae

**Affiliations:** University of South Florida, Tampa, Florida, United States; University of Missouri, Columbia, Missouri, United States; University of South Florida, Tampa, Florida, United States; University of South Florida, Tampa, Florida, United States; University of Missouri, Columbia, Missouri, United States; University of South Florida, Tampa, Florida, United States; University of South Florida, Tampa, Florida, United States

## Abstract

Informal caregiving is associated with sleep and stress, particularly among women, but no studies have examined the impact of caregiving for different individuals (child, parent, spouse) on sleep and stress. This study fills that gap. Caregivers of a child (N=151, Mage=40.50, 52%-female), parent (N=99, Mage=44.45, 52%-female), and spouse (N=71, Mage=55.26, 45%-female) completed the Perceived Stress Scale and reported bed/waketime consistency and night awakenings. Three-way ANOVAs evaluated interactions (caregiver-type x sleep/stress x sex) and main effects, controlling for age. Main effects also were examined separately for men and women. Pairwise comparisons clarified significant differences. Interactions not significant. A significant main effect (p=.049) occurred for night awakenings, consistent bedtime (p=.025), consistent waketime (p=.049), and stress (p=.045). Caregiver-child reported less consistent bedtimes (M=3.66+\-1.73) than caregiver-parent (M=2.93+\-1.63; p<.001) or caregiver-spouse (M=3.20+\-1.78; p=.009). Caregiver-child reported less consistent waketimes (M=3.23+\-1.62) than caregiver-parent (M=2.61+\-1.52; p=.003). Caregiver-child (M=4.39+\-2.87; p=.009) and caregiver-parent (M=4.28+\-2.91; p=.031) reported more night awakenings than caregiver-spouse (M=3.35+\-2.46). Caregiver-child reported more stress (M=19.91+\-5.95) than caregiver-spouse (M=17.56+\-7.46; p=.013). For women, a significant main effect (p=.008) occurred for stress and consistent waketimes (p=.047). Among women, caregiver-child reported more stress (M=21.25+\-6.02) than caregiver-parent (M=20.75+\-6.0; p=.014) or caregiver-spouse (M=16.63+\-7.4; p<.001). Caregiver-child reported less consistent waketimes for women (M=3.25+\-1.71) than caregiver-parent (M=2.83+\-1.30; p=.010). Thus, child and parent caregivers, particularly women, may experience increased sleep problems/stress. This may be in part due to caregiving burdens placed on women. Thus, women caregivers may need additional monitoring and support. We encourage longitudinal research examining what contributes to increased sleep and stress among caregivers.

